# Supplementary material for: Alzheimer’s Disease Risk Variants Interact with Amyloid-Beta to Modulate Monocyte Function
Source: Cells. 2025 Dec 15;14(24):1990. doi: 10.3390/cells14241990 (PMC12731656; doi:10.3390/cells14241990)
Supplement: Supplementary file 1 [file cells-14-01990-s001.zip › cells-3993740-supplementary.pptx]

## Slide 1
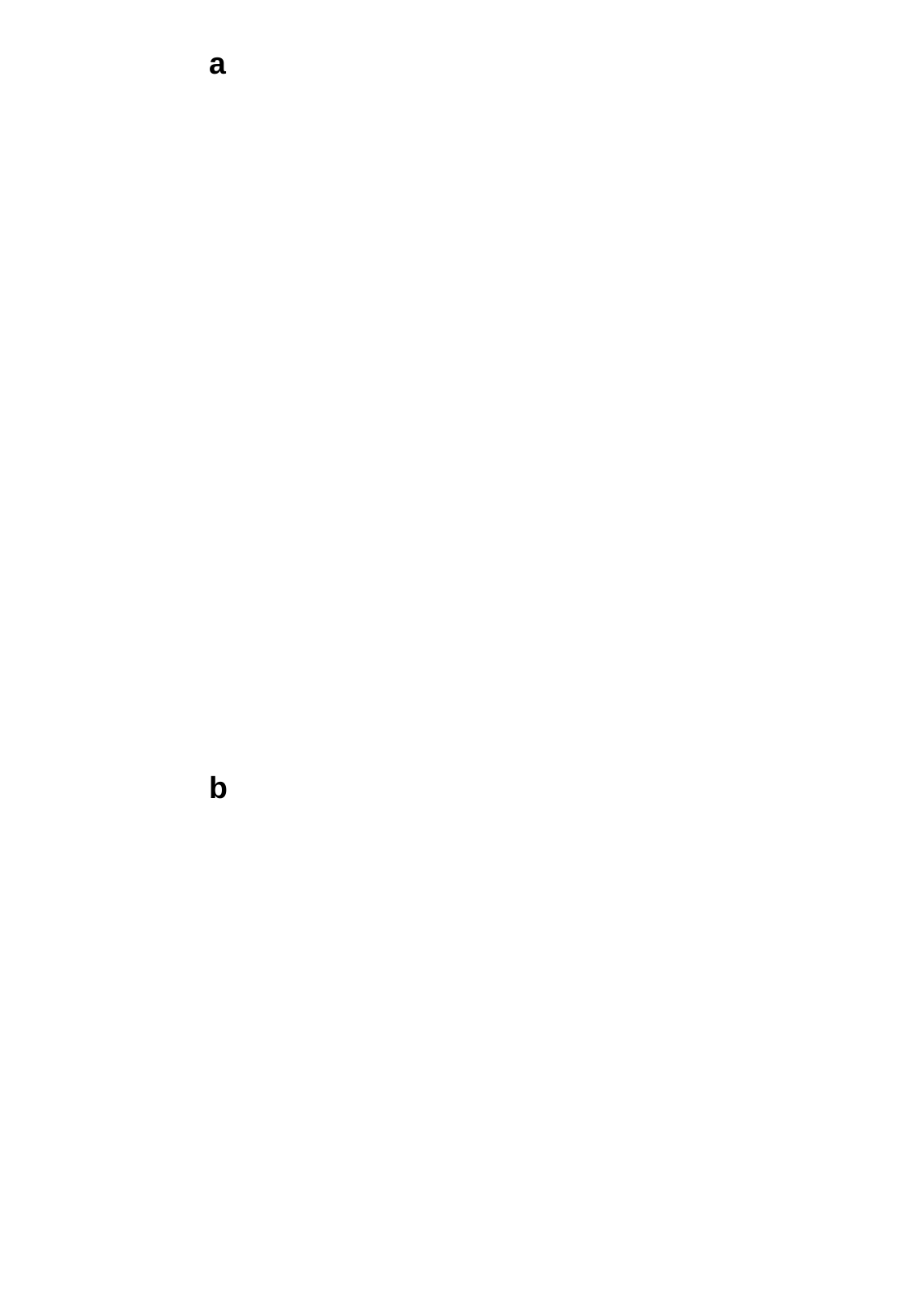

a
b

## Slide 2
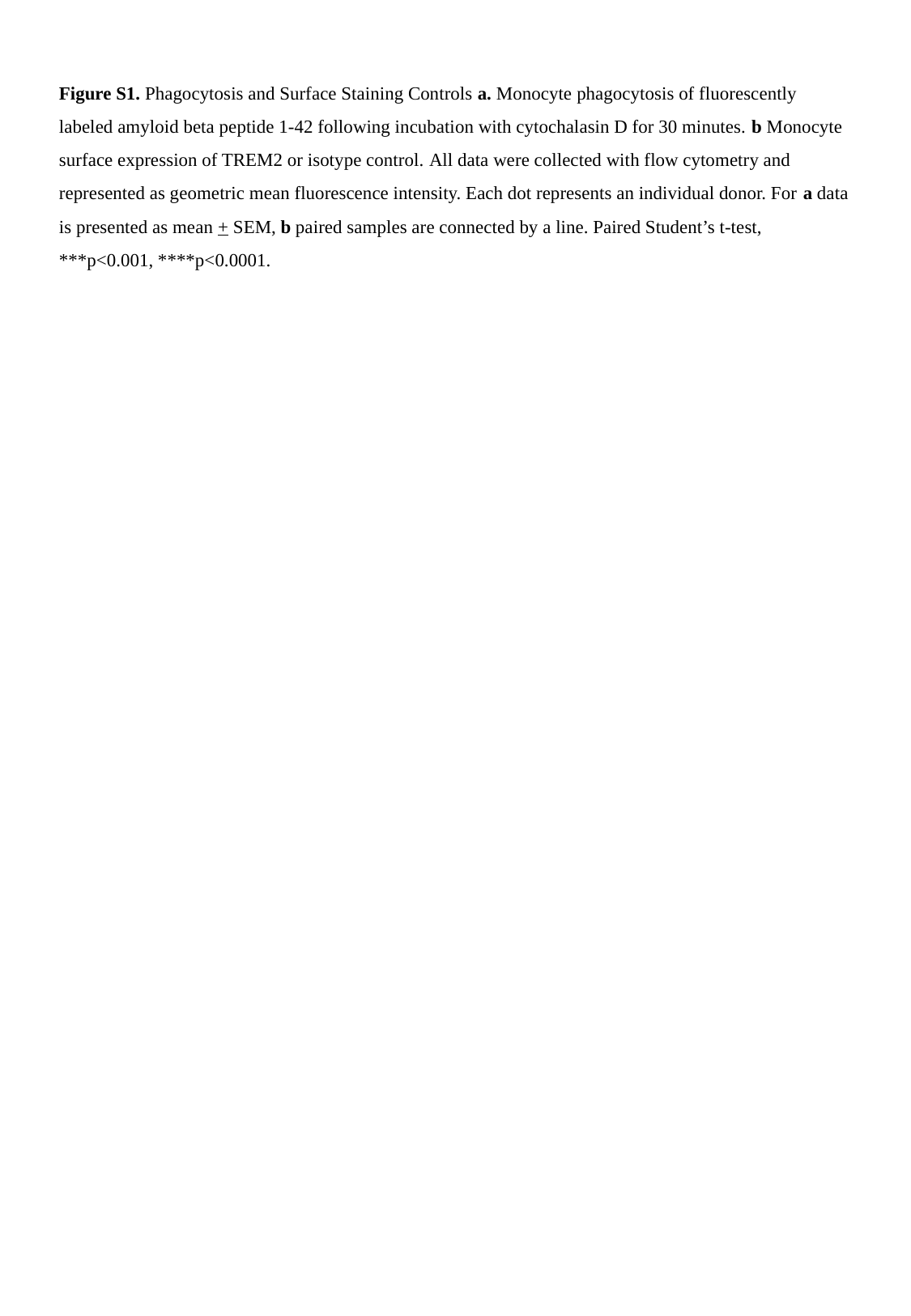

Figure S1. Phagocytosis and Surface Staining Controls a. Monocyte phagocytosis of fluorescently labeled amyloid beta peptide 1-42 following incubation with cytochalasin D for 30 minutes. b Monocyte surface expression of TREM2 or isotype control. All data were collected with flow cytometry and represented as geometric mean fluorescence intensity. Each dot represents an individual donor. For a data is presented as mean + SEM, b paired samples are connected by a line. Paired Student’s t-test, ***p<0.001, ****p<0.0001.

## Slide 3
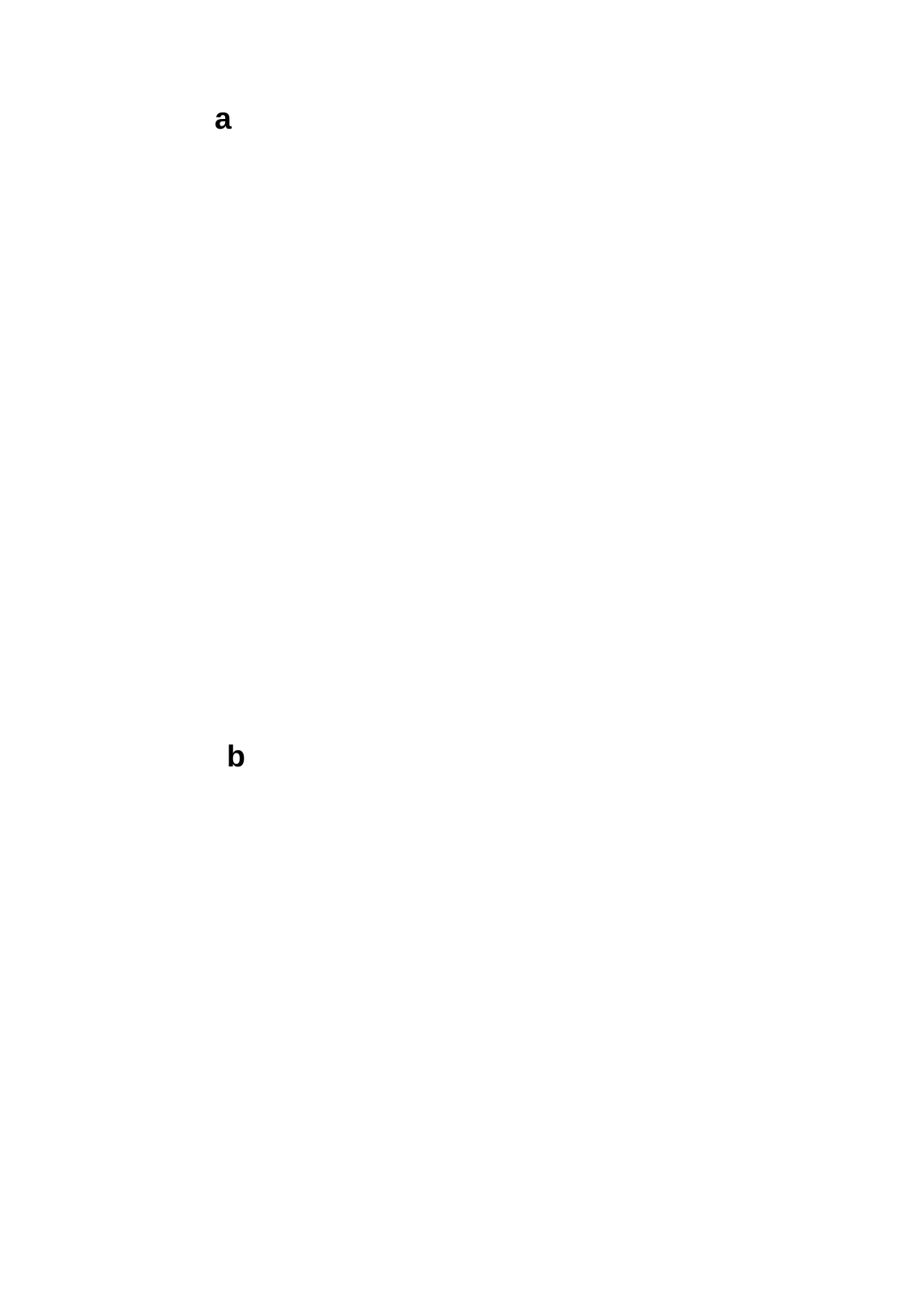

a
b

## Slide 4
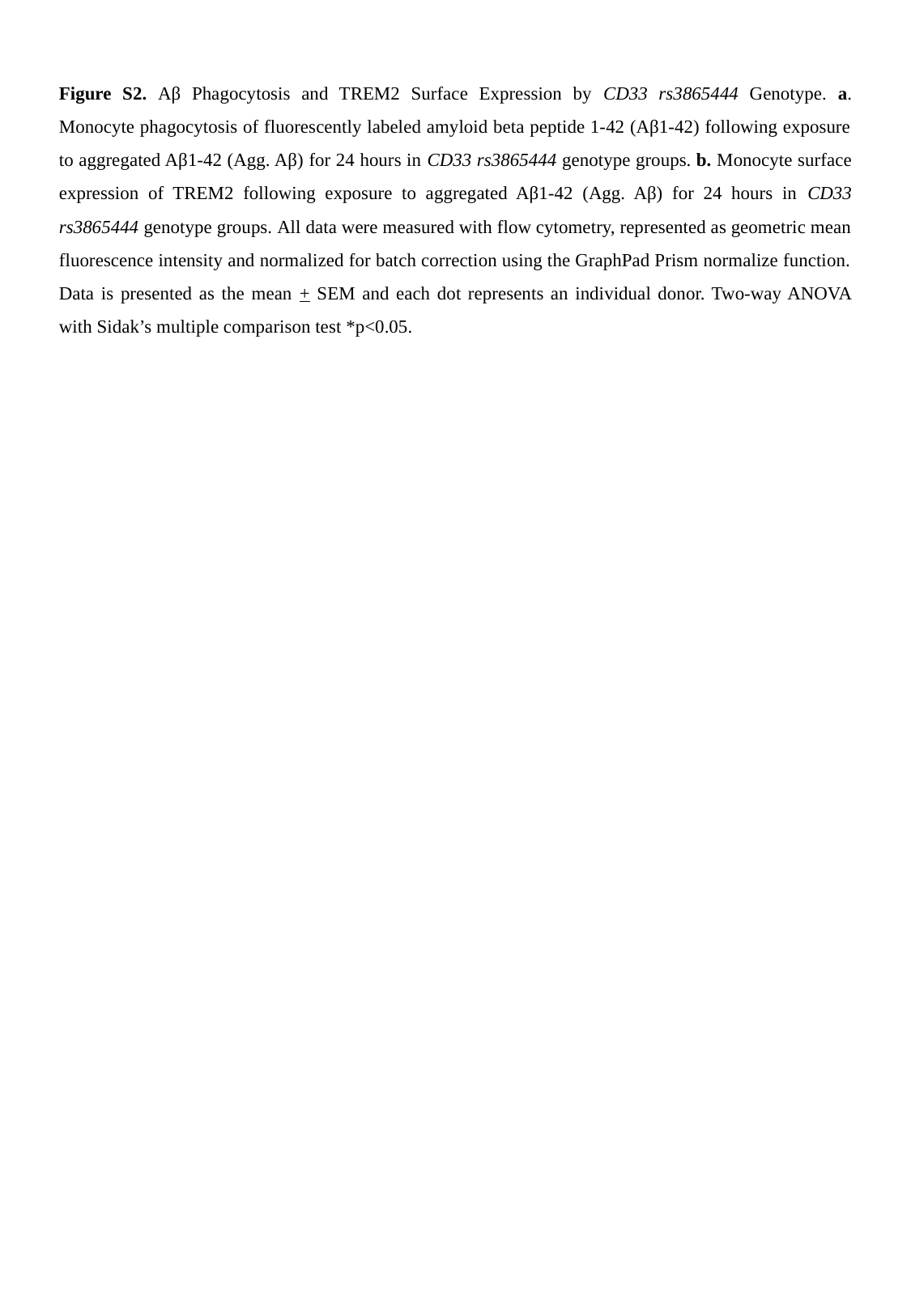

Figure S2. Aβ Phagocytosis and TREM2 Surface Expression by CD33 rs3865444 Genotype. a. Monocyte phagocytosis of fluorescently labeled amyloid beta peptide 1-42 (Aβ1-42) following exposure to aggregated Aβ1-42 (Agg. Aβ) for 24 hours in CD33 rs3865444 genotype groups. b. Monocyte surface expression of TREM2 following exposure to aggregated Aβ1-42 (Agg. Aβ) for 24 hours in CD33 rs3865444 genotype groups. All data were measured with flow cytometry, represented as geometric mean fluorescence intensity and normalized for batch correction using the GraphPad Prism normalize function. Data is presented as the mean + SEM and each dot represents an individual donor. Two-way ANOVA with Sidak’s multiple comparison test *p<0.05.

## Slide 5
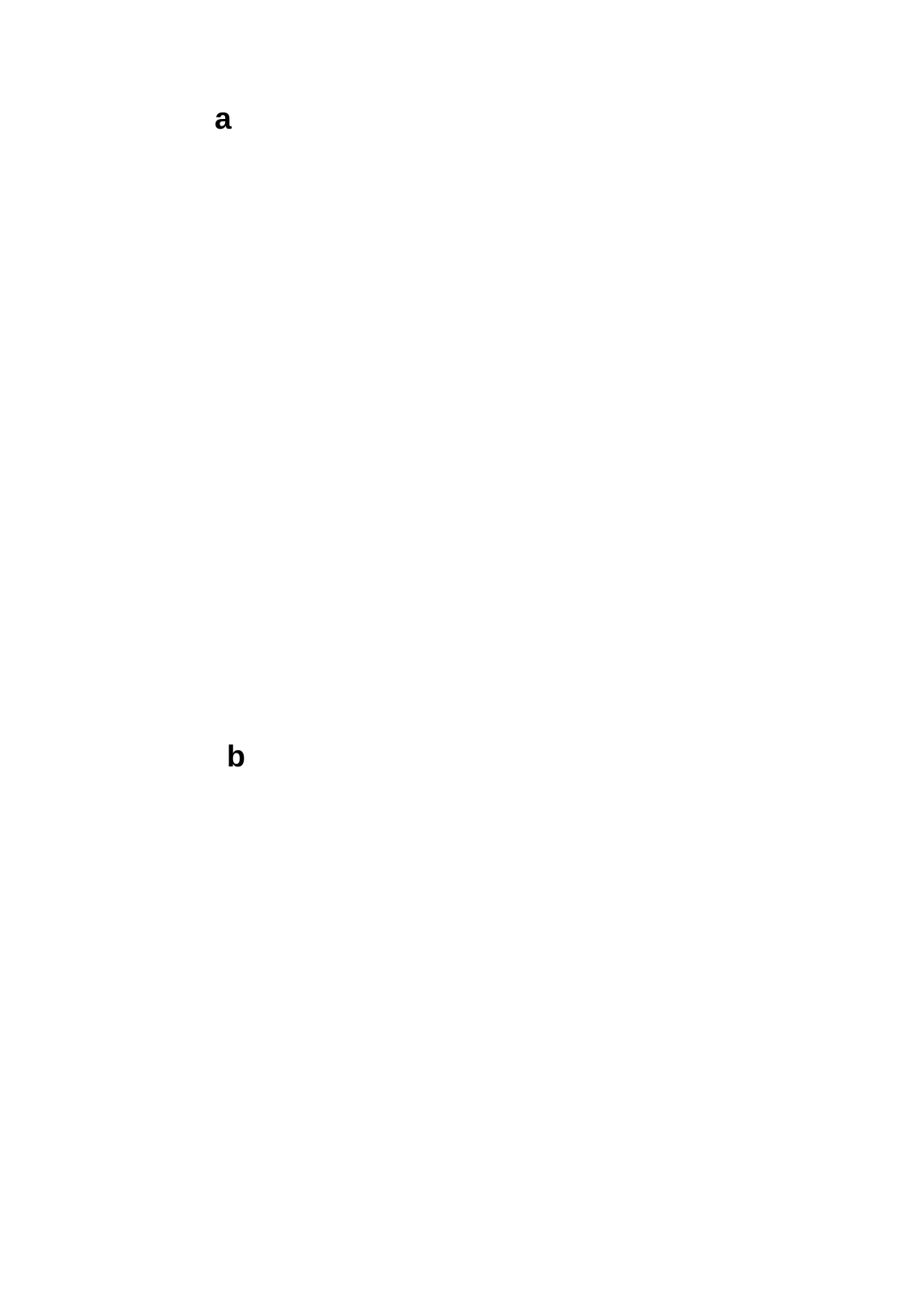

a
b

## Slide 6
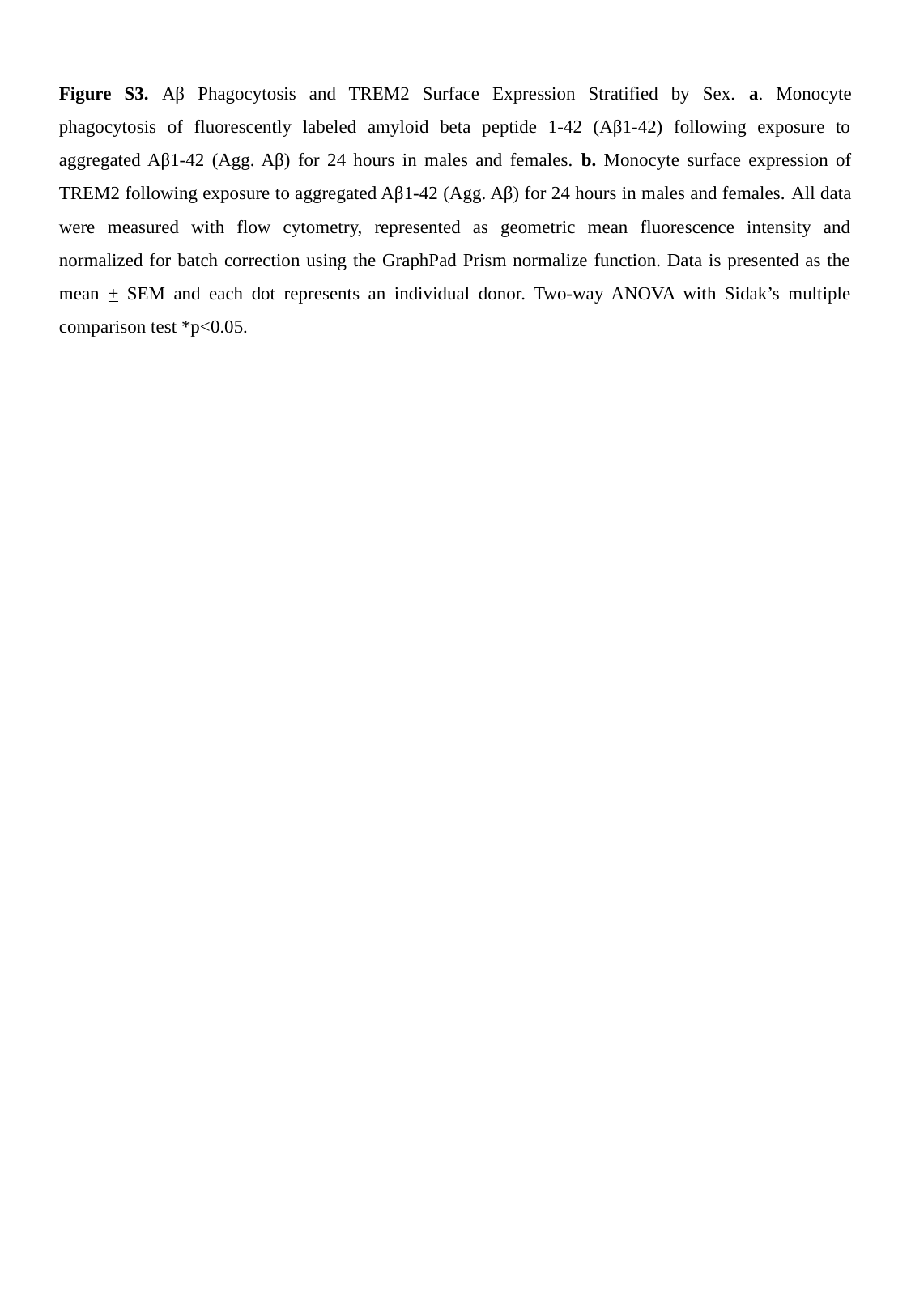

Figure S3. Aβ Phagocytosis and TREM2 Surface Expression Stratified by Sex. a. Monocyte phagocytosis of fluorescently labeled amyloid beta peptide 1-42 (Aβ1-42) following exposure to aggregated Aβ1-42 (Agg. Aβ) for 24 hours in males and females. b. Monocyte surface expression of TREM2 following exposure to aggregated Aβ1-42 (Agg. Aβ) for 24 hours in males and females. All data were measured with flow cytometry, represented as geometric mean fluorescence intensity and normalized for batch correction using the GraphPad Prism normalize function. Data is presented as the mean + SEM and each dot represents an individual donor. Two-way ANOVA with Sidak’s multiple comparison test *p<0.05.

## Slide 7
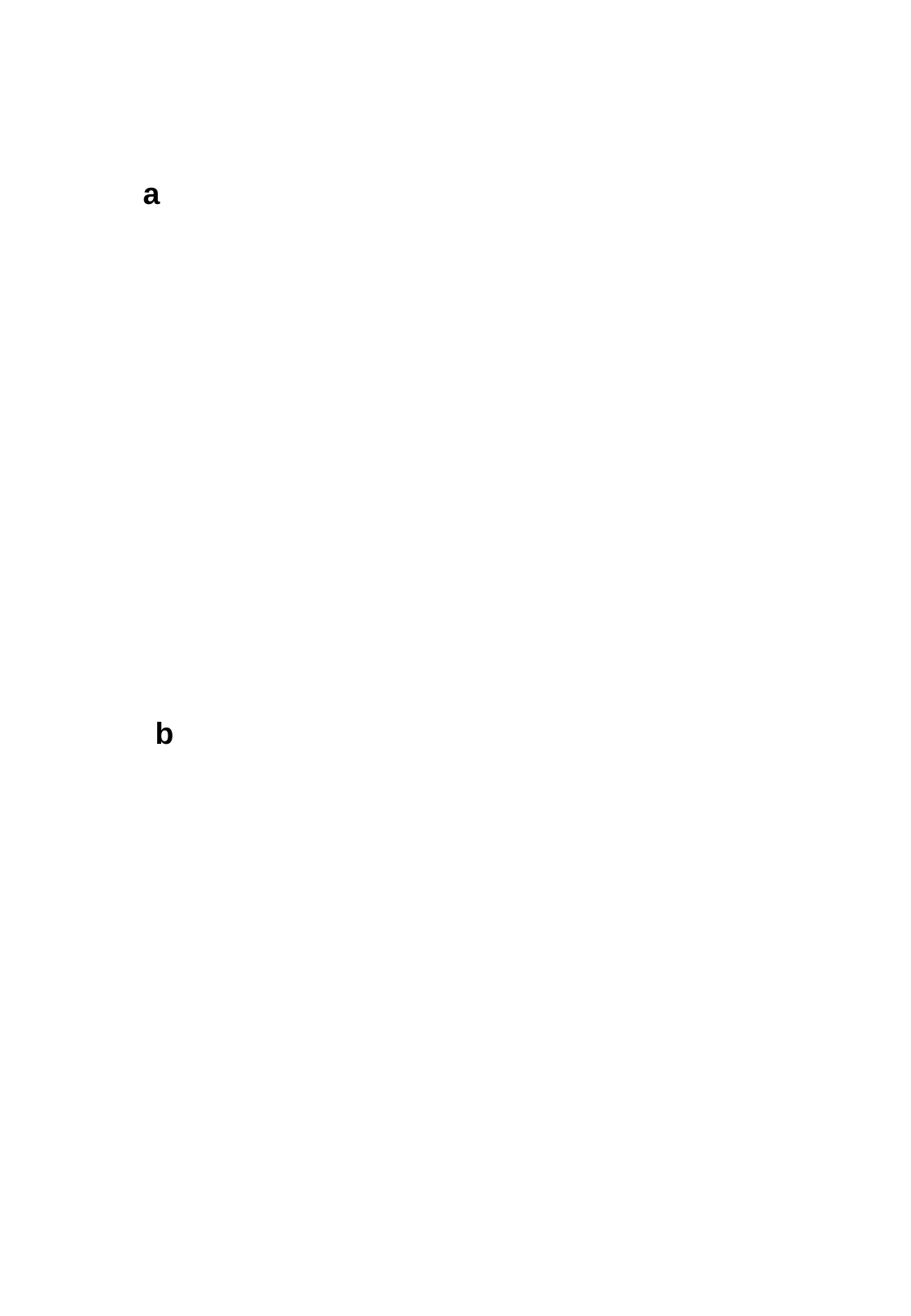

a
b

## Slide 8
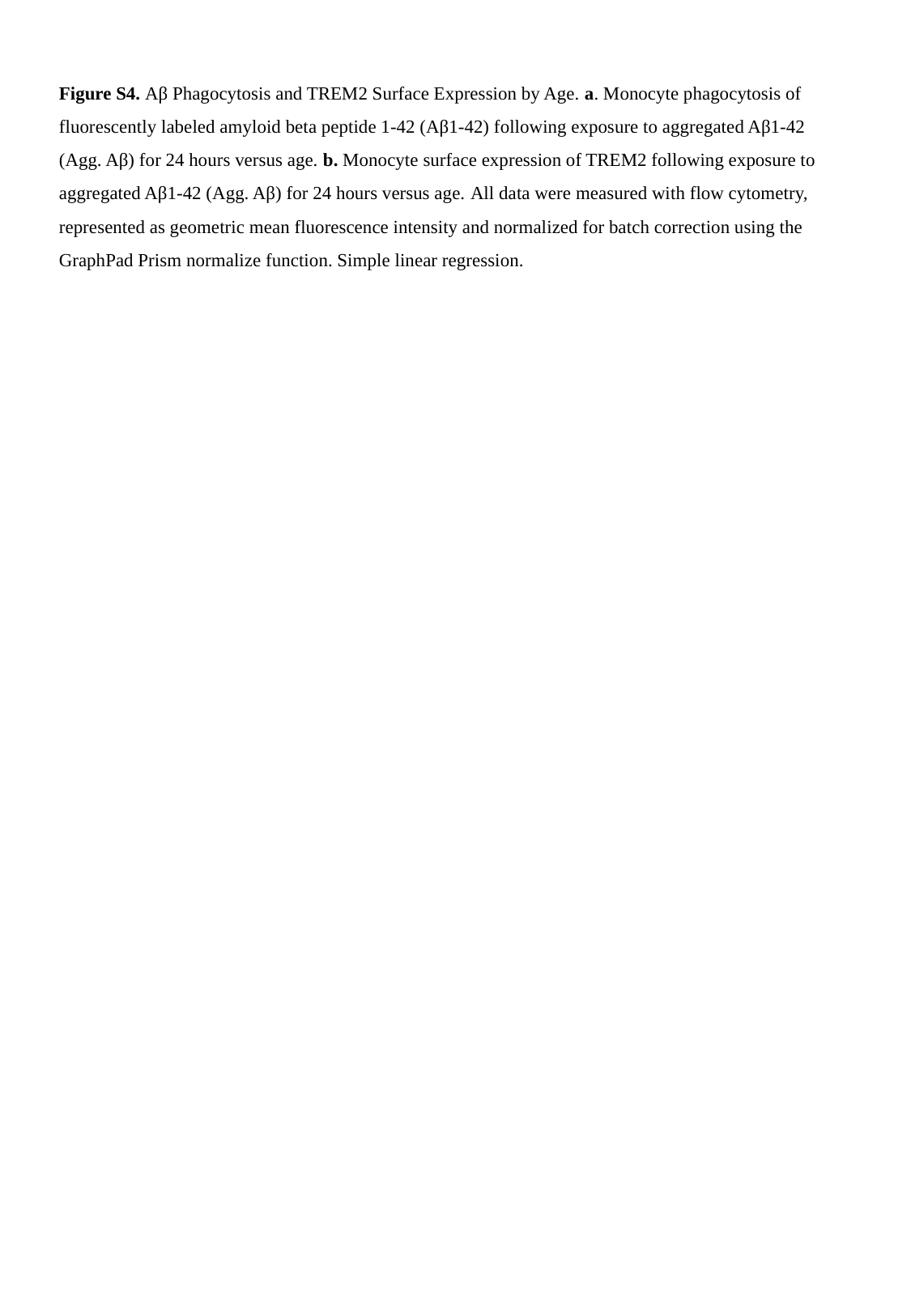

Figure S4. Aβ Phagocytosis and TREM2 Surface Expression by Age. a. Monocyte phagocytosis of fluorescently labeled amyloid beta peptide 1-42 (Aβ1-42) following exposure to aggregated Aβ1-42 (Agg. Aβ) for 24 hours versus age. b. Monocyte surface expression of TREM2 following exposure to aggregated Aβ1-42 (Agg. Aβ) for 24 hours versus age. All data were measured with flow cytometry, represented as geometric mean fluorescence intensity and normalized for batch correction using the GraphPad Prism normalize function. Simple linear regression.

## Slide 9
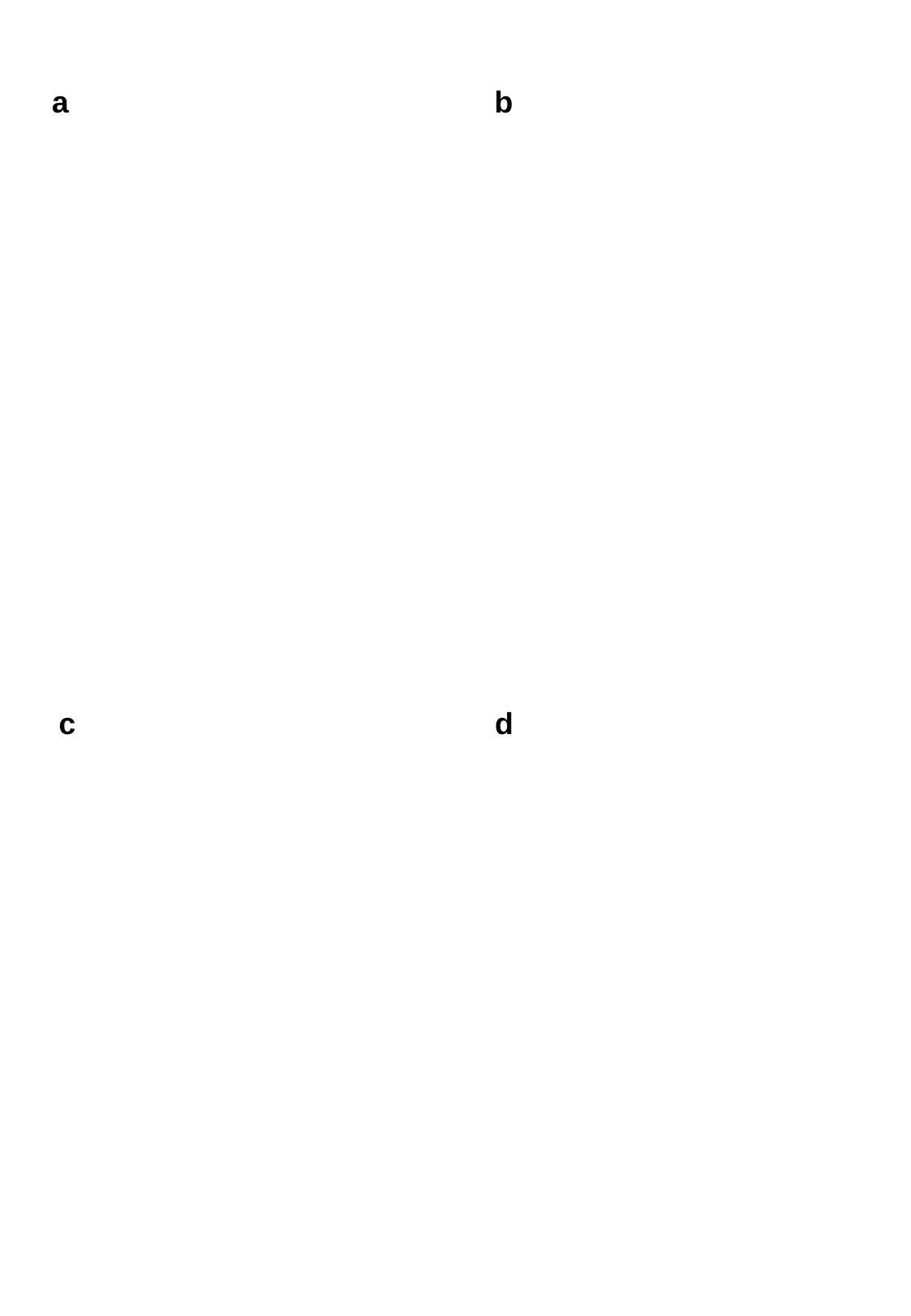

a
b
c
d

## Slide 10
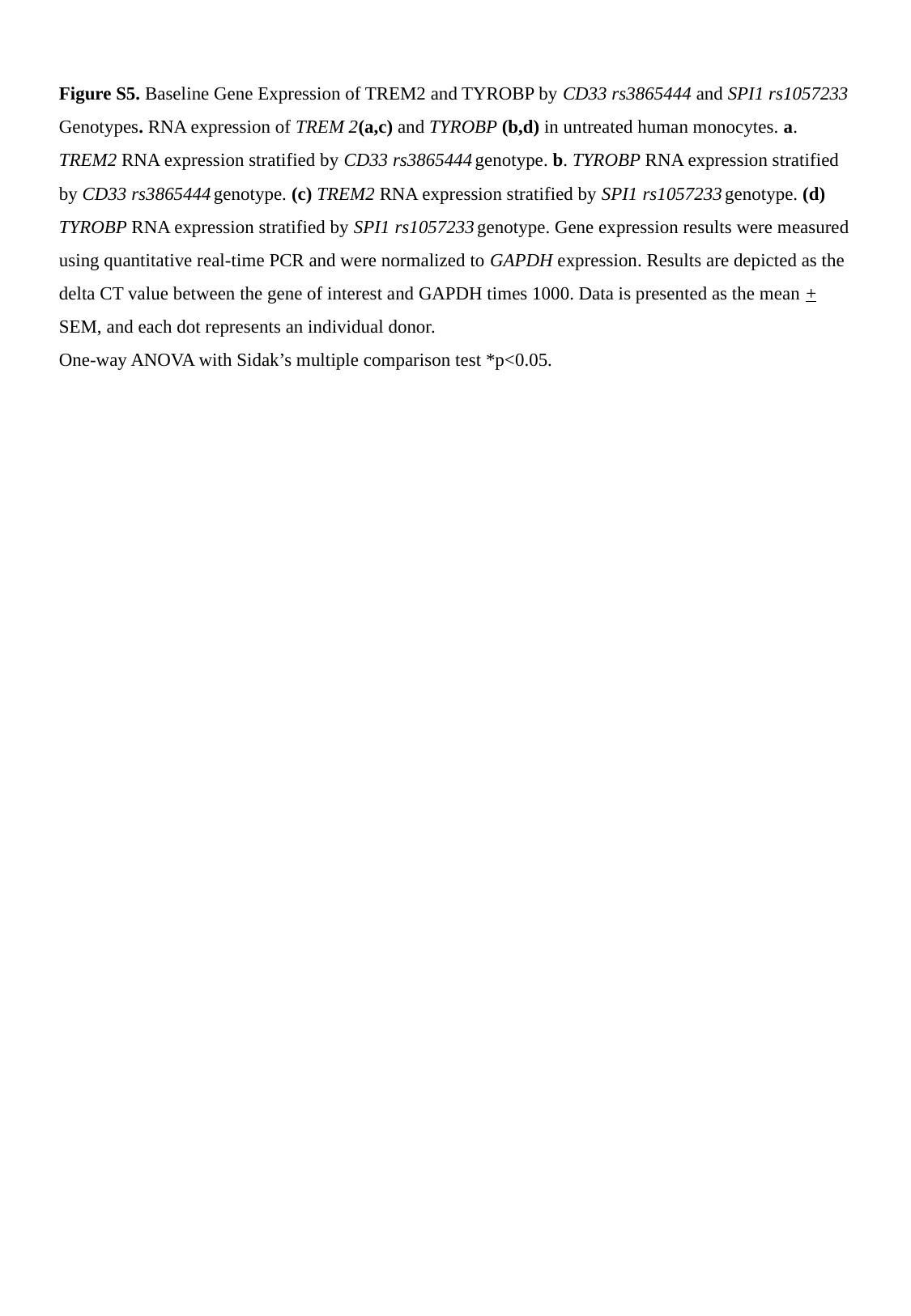

Figure S5. Baseline Gene Expression of TREM2 and TYROBP by CD33 rs3865444 and SPI1 rs1057233 Genotypes. RNA expression of TREM 2(a,c) and TYROBP (b,d) in untreated human monocytes. a. TREM2 RNA expression stratified by CD33 rs3865444 genotype. b. TYROBP RNA expression stratified by CD33 rs3865444 genotype. (c) TREM2 RNA expression stratified by SPI1 rs1057233 genotype. (d) TYROBP RNA expression stratified by SPI1 rs1057233 genotype. Gene expression results were measured using quantitative real-time PCR and were normalized to GAPDH expression. Results are depicted as the delta CT value between the gene of interest and GAPDH times 1000. Data is presented as the mean + SEM, and each dot represents an individual donor.
One-way ANOVA with Sidak’s multiple comparison test *p<0.05.
